# Real-world characteristics of systemic mastocytosis in Romania: insights from a reference-center-based descriptive study

**DOI:** 10.25122/jml-2025-0103

**Published:** 2025-07

**Authors:** Delia Soare, Dan Soare, Brîndușa Petruțescu, Oana Firescu, Poliana Leru, Corina Silvia Pop, Lucian Negreanu, Vlad Voiculescu, Horia Bumbea

**Affiliations:** 1 Bone Marrow Transplantation Unit, University Emergency Hospital Bucharest, Bucharest, Romania; 2 Scientific Research Methodology and Hematology, Carol Davila University of Medicine and Pharmacy, Bucharest, Romania; 3 Allergology, University Emergency Hospital Bucharest, Bucharest, Romania; 4 Dermatology, University Emergency Hospital Bucharest, Bucharest, Romania; 5 Allergology Department, Colentina Clinical Hospital, Bucharest, Romania; 6 Clinical Department 5, Carol Davila University of Medicine and Pharmacy, Bucharest, Romania; 7 Gastroenterology II, University Emergency Hospital Bucharest, Bucharest, Romania; 8 Internal Medicine III and Gastroenterology, Carol Davila University of Medicine and Pharmacy, Bucharest, Romania; 9 Gastroenterology I, University Emergency Hospital Bucharest, Bucharest, Romania; 10 Internal Medicine I and Gastroenterology, Carol Davila University of Medicine and Pharmacy, Bucharest, Romania; 11 Oncological Dermatology, Carol Davila University of Medicine and Pharmacy, Bucharest, Romania; 12 Department of Dermatology and Allergology, Elias Emergency University Hospital, Bucharest, Romania

**Keywords:** systemic mastocytosis, Romania, epidemiology, *KIT* D816V mutation, clinical phenotype, targeted therapy, SM, Systemic Mastocytosis, ASM, Aggressive Systemic Mastocytosis, ISM, Indolent Systemic Mastocytosis, SSM, Smoldering Systemic Mastocytosis, SM-AHN, Systemic Mastocytosis with Associated Hematologic Neoplasm, CM, Cutaneous Mastocytosis, MCL, Mast Cell Leukemia, WDSM, Well-Differentiated Systemic Mastocytosis, SM-ana, Systemic Mastocytosis without skin involvement associated with recurrent anaphylaxis or vascular collapse, KIT proto-oncogene, receptor tyrosine kinase, TKIs, Tyrosine Kinase Inhibitors, FLT3, FMS-like Tyrosine Kinase 3, WHO, World Health Organization, IWG-MRT-ECNM, International Working Group – Myeloproliferative Neoplasms Research & Treatment – European Competence Network on Mastocytosis, MRI, Magnetic Resonance Imaging

## Abstract

Systemic mastocytosis (SM) is a rare clonal mast cell disease characterized by heterogeneous clinical presentations and molecular features that vary across different regions; however, data from Central-Eastern Europe remain limited. This study aimed to describe the demographic, clinical, laboratory, and molecular characteristics of Romanian adults diagnosed with SM and followed at the national reference center for mast cell disorders in Bucharest, while also exploring real-world management patterns and outcomes. We conducted a retrospective observational study including 162 adult patients evaluated between January 2006 and March 2025 who met the 2022 World Health Organization criteria for SM. Data extracted from electronic medical records included WHO subtype, *KIT* mutation status, serum tryptase levels, organ involvement, treatment history, and survival outcomes. A descriptive statistical approach was used, with continuous variables expressed as medians and interquartile ranges, and categorical variables as counts and percentages. A subset of eight patients underwent bone marrow flow cytometry immunophenotyping to assess the diagnostic contribution of CD2 and CD25. Among the entire cohort, indolent SM was the most frequent form (53.1%), followed by cutaneous mastocytosis (17.3%), smoldering SM (5.6%), aggressive SM (3.7%), and SM with associated hematologic neoplasm (8%). The *KIT* D816V mutation was detected in 43% of tested individuals, and the median baseline serum tryptase was 20.55 μg/L. Common organ-related findings included osteoporosis (17.9%), osteopenia (21.7%), cutaneous lesions (29%), and hepatomegaly (4%). First-line symptomatic therapy (H_1_/H_2_ antihistamines ± montelukast) was administered to 77% of patients, while four patients with advanced disease received midostaurin treatment. Immunophenotyping confirmed aberrant expression of CD2 and CD25 in all eight analyzed cases. This first national series from Romania underscores the predominance of indolent SM and the clinical burden of organ involvement, reinforcing the need for early diagnosis and personalized, risk-adapted therapeutic approaches.

## INTRODUCTION

Systemic mastocytosis (SM) is a clonal mast-cell neoplasm that encompasses a spectrum of indolent to highly aggressive variants, newly codified in the 5^th^ edition of the World Health Organization (WHO) classification of haematolymphoid tumours (2022) [1]. Although considered a rare disease, its population burden is non-trivial: recent nationwide data from Sweden place the annual adult incidence at ≈1.6 per 100 000 and the prevalence near 24 per 100 000 [2], while regional Italian estimates are comparable (incidence 1.1 and prevalence 10–17 per 100 000) [3]. Geography appears to influence both epidemiology and phenotype; yet, Central-Eastern Europe remains underrepresented in large datasets [2,4,5].

Pathobiologically, more than 90% of adult SM cases harbor the constitutively activating *KIT* D816V mutation, which drives mast cell expansion and mediator release [6,7]. The mutation has become a therapeutic target: the multikinase inhibitor midostaurin improved response and survival in advanced SM and gained regulatory approval in 2017 [8,9], and the selective KIT D816V inhibitor avapritinib has recently shown durable efficacy in the phase 2 PATHFINDER trial, prompting approvals in Europe and North America [10]. Despite these advances, outcomes for aggressive subtypes and mast-cell leukaemia remain poor, and access to targeted agents is uneven across Europe.

Large-scale real-world data, primarily generated through the European Competence Network on Mastocytosis (ECNM) registry, have refined prognostic scoring and highlighted the value of multidisciplinary centres of expertise [11]. However, published ECNM cohorts include few or no Romanian patients, and national epidemiology, molecular landscape, and management patterns are essentially unknown. This knowledge gap hampers benchmarking of local practice against European standards and may delay the adoption of novel therapies.

Under normal conditions, mast cells are rare in the bone marrow, comprising less than 1% of marrow elements. Their characteristic immunophenotype includes strong expression of CD117, intermediate expression of CD33, CD9, and CD71, low levels of CD38 and CD11b, and no expression of CD34 or CD19 [12-14]. This phenotype places them near eosinophils on CD45/side-scatter plots and distinguishes them from other hematopoietic cells [12].

In systemic mastocytosis (SM), mast cells exhibit aberrant immunophenotypes that can be reliably detected by flow cytometric immunophenotyping (FCI). Despite its diagnostic value, FCI is not widely used in the routine workup of SM due to technical challenges and limited awareness of its utility in mast cell analysis. Aberrant expression of CD25, with or without CD2, is considered a minor diagnostic criterion by the 2022 WHO classification [1]. In our practice, a multiparametric flow cytometry (MFC) panel incorporating markers such as CD2, CD25, and CD30 has been implemented. This assay proves especially useful in suspected or referred SM cases, in the presence of eosinophilia, or when mast cells are incidentally increased, providing an early clue to clonal eosinophilic disorders or myeloid/lymphoid neoplasms with eosinophilia and tyrosine kinase fusions.

Here we present the first reference–center–based descriptive study of Romanian adults with SM. We analyse demographics, clinical and molecular features, treatment pathways for consecutive patients evaluated at the national Centre of Expertise in Bucharest over 19 years. By comparing our findings with pan-European cohorts, we aim to: (i) characterise the real-world Romanian SM population, (ii) identify unmet clinical needs, and (iii) provide a baseline against which future therapeutic interventions can be measured. Our results confirm a predominance of indolent disease yet reveal a substantial burden of organ damage and limited access to diagnostic procedures, underscoring the importance of early referral and centre-based multidisciplinary care.

## MATERIAL AND METHODS

### Study design and setting

We conducted a retrospective, observational cohort study at the Reference Center for Mast Cell Disorders, University Emergency Hospital Bucharest. Adults diagnosed with systemic mastocytosis between January 2006 and March 2025 were reviewed.

### Participants

A total of 162 patients met all inclusion criteria: (i) clinical presentation suggestive of SM, (ii) available baseline serum tryptase, and (iii) available *KIT* mutation testing. Exclusion criteria were: age < 18 years; absence of confirmatory bone-marrow histology; incomplete core variables; duplicate records; withdrawal of informed consent; or an external SM diagnosis lacking source documentation for internal verification.

### Data sources

Demographic, clinical, laboratory, and treatment data were extracted from the hospital’s electronic medical record system, the center’s dedicated SM registry, and archived paper charts. All entries were cross-checked by two investigators.

### Diagnostic procedures

SM was confirmed according to the 2022 WHO criteria through an iliac-crest bone marrow biopsy with haematoxylin-eosin staining, mast cell tryptase immunohistochemistry, and, when required, flow cytometric immunophenotyping of marrow aspirates. Molecular testing for *KIT* D816V (allele-specific PCR) was performed at the molecular pathology laboratory of the Emergency University Hospital Bucharest. Baseline evaluation included serum tryptase, dual-energy X-ray absorptiometry, contrast-enhanced computed tomography (CT) or magnetic resonance imaging (MRI), abdominal ultrasonography, and upper gastrointestinal endoscopy, as clinically indicated. Limited reimbursement and reagent shortages restricted timely access to flow cytometry and extended *KIT* sequencing.

### Treatment documentation and follow-up

Pharmacologic management (H1/H2-antihistamines, leukotriene antagonists, and midostaurin) and supportive therapies were documented anamnestically and verified against pharmacy records. Vital status and date of last contact were updated to 31 March 2025.

### Outcomes

Primary endpoints were the distribution of WHO SM subtypes and overall survival (OS). Secondary endpoints included the frequencies of organ involvement and treatment patterns.

### Flow cytometry

We analyzed eight bone marrow aspirates from patients diagnosed with either indolent or advanced SM according to WHO criteria, using flow cytometric immunophenotyping to characterize the mast cell phenotype. The samples were stained with monoclonal antibodies targeting surface antigens commonly associated with neoplastic mast cells, including CD2, CD25, CD35, CD59, CD63, and CD69, as previously described [15,16]. To detect CD2 and CD25 expression, both PE- and FITC-conjugated antibodies were tested. High CD117 expression was used as a gating strategy to isolate mast cells, while myeloid precursors (CD34+CD117+) were excluded. Data acquisition was performed using the FACS Canto II flow cytometer in the Stem Cell Laboratory at the University Emergency Hospital Bucharest. Analysis was conducted using Infinicyte software version 2.0, Cytognos SL.

### Statistical analysis

All analyses were conducted using SPSS version 29 (IBM Corp.) and JASP (Just Another Statistical Program), version 0.19.3. Continuous variables are reported as median (interquartile range, IQR) and compared using the Mann-Whitney U test or the Kruskal–Wallis test. Categorical variables are reported as counts (%) and compared using the chi-square (χ^2^) test or Fisher’s exact test. Two-sided *P* values < 0.05 were considered statistically significant.

## RESULTS

### Demographic data and patient characteristics

All patients were divided into two groups according to sex, and a statistically significant difference was observed in distribution, with approximately twice as many women as men. Age at diagnosis and current age were also analyzed based on sex (Table 1).

**Table 1:** Demographic and biological characteristics of patients divided into two groups based on gender

PARAMETERS	Gender				Statistical significance
	Female		Male		
	*n* = 106		*n* = 56		
	Mean ± Standard deviation	Median	Mean ± Standard deviation	Median	*P* value
Age at diagnosis (years)	44.30 ± 11.06	43	42.36 ± 15.42	41	0.407
Current age (Years)	50.41 ± 11.53	49	46.15 ± 15.05	45	0.048
Tryptase level (µg/L)	42.35 ± 72.06	22.8	30.17 ± 35.84	18.3	0.246
LDH (U/L)	198.88 ± 56.58	190.5	206.15 ± 72.24	201	0.486
Bone marrow infiltrate (%)	15.73 ± 12.94	15	14.81 ± 11.75	15	0.277
Sodium cromoglycate dosage (mg)	806.35 ± 423.07	800	958.06 ± 446.30	800	0.112
Alkaline phosphatase (U/L)	88.85 ± 67.99	76	87.53 ± 38.77	76	0.896

The median age at diagnosis was 43 years in the female group and 41 years in the male group, with no statistically significant difference (*P* = 0.407). In contrast, the current age showed a statistically significant difference, with a median of 49 years in the female group and 45 years in the male group (*P* = 0.048).

Although the difference in serum tryptase levels between the two groups was not statistically significant, the median values in both sexes exceeded the normal range. Notably, in the female group, the median tryptase level was above the minor diagnostic criterion (22.8 ng/mL vs. 20 ng/mL), while in the male group, it was slightly below this threshold (18.3 ng/mL vs. 20 ng/mL).

Using the same stratification by sex, serum lactate dehydrogenase (LDH) levels at the time of diagnosis were also analyzed. The median LDH level was 190.5 U/L in female participants and 201 U/L in male participants, with no statistically significant difference (*P* = 0.486). Additionally, the percentage of bone marrow infiltration was assessed, with both groups showing a median of 15% mast cell infiltration, defined according to the major diagnostic criterion as multifocal clusters of abnormal mast cells (more than 15 mast cells per aggregate) in the bone marrow or other extracutaneous tissues. This finding was also not statistically significant (*P* = 0.277).

The need for supportive therapy was also evaluated by analyzing the required daily dose of sodium cromoglycate used to manage gastrointestinal and cutaneous symptoms. Both sex groups had a comparable median dose of 800 mg/day, with no statistically significant difference (*P* = 0.112).

Finally, serum alkaline phosphatase (ALP) levels at diagnosis were analyzed by sex. The median ALP level was 76 U/L in both groups, with no statistically significant difference observed (*P* = 0.896).

Regarding bone involvement, the study population was stratified by sex. In the female group (*n* = 106), patients were evaluated for the presence of osteopenia or osteoporosis. Among them, 19 patients (17.9%) were diagnosed with osteoporosis, 23 (21.7%) with osteopenia, while 64 (60.4%) showed no evidence of bone involvement. In the male group (*n* = 56), four patients (17.4%) were diagnosed with osteoporosis, 11 (32.4%) with osteopenia, and 41 (39.0%) had no signs of bone involvement.

### Bivariate correlations

A comprehensive bivariate correlation analysis was conducted to explore associations between clinical variables and disease subtypes in the study cohort.

Cutaneous mastocytosis (CM) showed a moderate, statistically significant positive correlation with serum tryptase levels (Spearman’s rho = 0.403, *P* < .001; Pearson’s r = 0.208, *P* < .001), as well as with the percentage of bone marrow infiltration (Spearman’s rho = 0.364, *P* < .001; Pearson’s r = 0.314, *P* < .001). In contrast, CM was negatively correlated with symptomatic treatment (Spearman’s rho = -0.296, *P* < .001; Pearson’s r = -0.296, *P* < .001) and with the use of antihistamines (Spearman’s rho = -0.266, *P* < .001; Pearson’s r = -0.266, *P* < .001).

For indolent systemic mastocytosis (ISM), a moderate inverse correlation with serum tryptase levels was observed (Spearman’s rho = -0.388, *P* < .001). ISM showed a weak but significant positive correlation with bone involvement (Spearman’s rho = 0.189, *P* = 0.016; Pearson’s r = 0.186, *P* = 0.017) and a weak inverse correlation with *KIT* D816V mutation presence (Spearman’s rho = -0.187, *P* = 0.017; Pearson’s r = -0.148, *P* = 0.061). Additionally, ISM correlated inversely with the percentage of bone marrow infiltration (Spearman’s rho = -0.213, *P* = 0.019). A significant positive correlation was observed between ISM and the use of symptomatic treatment (Spearman’s rho = 0.454, *P* < .001; Pearson’s r = 0.454, *P* < .001), as well as with antihistamine use (Spearman’s rho = 0.413, *P* < .001; Pearson’s r = 0.413, *P* < .001).

In smoldering systemic mastocytosis (SSM), a moderate inverse correlation was observed with serum tryptase levels (Spearman’s rho = -0.303, *P* < .001; Pearson’s r = -0.398, *P* < .001). A weak positive correlation was found with bone involvement (Spearman’s rho = 0.168, *P* = 0.033; Pearson’s r = 0.168, *P* = 0.033). Moderate positive correlations were observed between SSM and hepatomegaly (Spearman’s rho = 0.388, *P* < .001; Pearson’s r = 0.388, *P* < .001) and with splenomegaly (Spearman’s rho = 0.387, *P* < .001; Pearson’s r = 0.387, *P* < .001). A significant inverse correlation was found between SSM and bone marrow infiltration (Spearman’s rho = -0.379, *P* < .001; Pearson’s r = -0.471, *P* < .001).

Aggressive systemic mastocytosis (ASM) was moderately positively correlated with the use of cladribine (Spearman’s rho = 0.343, *P* < .001; Pearson’s r = 0.343, *P* < .001) and imatinib (Spearman’s rho = 0.274, *P* < .001; Pearson’s r = 0.274, *P* < .001). A strong inverse correlation was observed between ASM and serum tryptase (Spearman’s rho = -0.281, *P* < .001; Pearson’s r = -0.579, *P* < .001). ASM was also significantly correlated with bone involvement (Spearman’s rho = 0.159, *P* = 0.043; Pearson’s r = 0.181, *P* = 0.021), hepatomegaly (Spearman’s rho = 0.256, *P* < .001; Pearson’s r = 0.256, *P* < .001), and splenomegaly (Spearman’s rho = 0.411, *P* < .001; Pearson’s r = 0.411, *P* < .001). An inverse correlation was observed between ASM and the percentage of bone marrow infiltration (Spearman’s rho = -0.334, *P* < .001; Pearson’s r = -0.405, *P* < .001), and also with serum alkaline phosphatase levels (Spearman’s rho = -0.275, *P* < .001; Pearson’s r = -0.477, *P* < .001).

In SM with associated hematologic neoplasm (SM-AHN), a statistically significant inverse correlation was found with cutaneous involvement (Spearman’s rho = -0.320, *P* < .001; Pearson’s r = -0.320, *P* < .001).

Further bivariate analyses showed that serum tryptase was inversely correlated with hepatomegaly (Spearman’s rho = -0.335, *P* < .001; Pearson’s r = -0.314, *P* < .001) and splenomegaly (Spearman’s rho = -0.294, *P* < .001; Pearson’s r = -0.449, *P* < .001). Tryptase levels correlated positively with the percentage of bone marrow infiltration (Spearman’s rho = 0.631, *P* < .001; Pearson’s r = 0.608, *P* < .001) and with serum alkaline phosphatase levels (Spearman’s rho = 0.311, *P* < .001; Pearson’s r = 0.563, *P* < .001).

**Figure 1:**
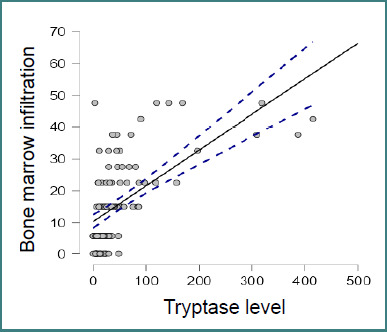
Bivariate correlation between tryptase level and bone marrow infiltration, Spearman’s rho = -0.376, *P* < 0.001

A significant inverse correlation was observed between bone involvement and the degree of bone marrow infiltration (Spearman’s rho = -0.376, *P* < .001; Pearson’s r = -0.358, *P* < .001; Figure 1. Likewise, bone marrow infiltration showed significant inverse correlations with symptomatic treatment (Spearman’s rho = -0.361, *P* < .001; Pearson’s r = -0.322, *P* < .001) and with antihistamine use (Spearman’s rho = -0.320, *P* < .001; Pearson’s r = -0.293, *P* = 0.001). In contrast, bone marrow infiltration showed a significant positive correlation with serum alkaline phosphatase levels (Spearman’s rho = 0.375, *P* < .001; Pearson’s r = 0.316, *P* < .001). Finally, a strong positive correlation was observed between symptomatic treatment and antihistamine use (Spearman’s rho = 0.841, *P* < .001; Pearson’s r = 0.841, *P* < .001).

**Figure 2:**
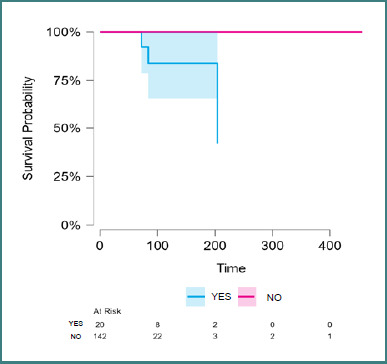
Kaplan-Meier survival curve comparing patients who received cytotoxic treatment vs the non-cytotoxic treatment group

A Kaplan-Meier survival analysis was conducted to compare outcomes between patients who received cytotoxic therapy and those who did not (Figure 2). The group receiving cytotoxic treatment included 20 patients, of whom three experienced events (deaths). This group had a median survival of 204 days and a restricted mean survival of 289.5 days (standard error: 79.25 days). In contrast, the non-cytotoxic treatment group included 142 patients, with no recorded events, and an estimated restricted mean survival of 456 days.

The Log-rank (Mantel-Haenszel) test demonstrated a statistically significant difference in survival curves (χ^2^ = 8.051; *P* = 0.005), indicating a negative impact of cytotoxic treatment on overall survival in this cohort. These findings were supported by the Peto and Fleming-Harrington tests, both of which yielded *P* values of 0.004.

### Flow cytometry findings

**Figure 3:**
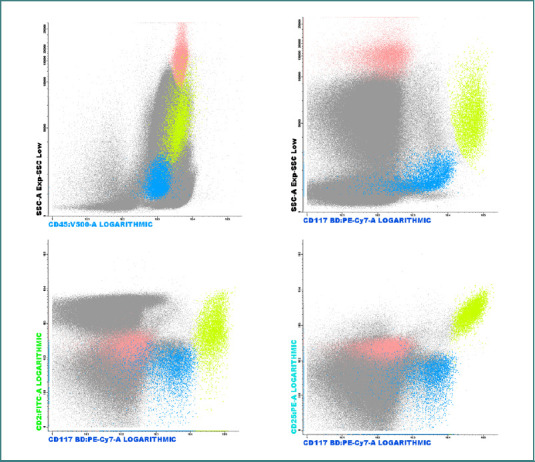
Dot-plot histograms illustrating the identification of myeloid precursor populations CD34+ CD117+ (blue), mast cells CD117++ CD34− (yellow), eosinophils with high SSC and high CD45 expression (pink), and the expression of CD2 and CD25 on mast cells (histograms 3 and 4)

We analyzed eight patients diagnosed with advanced or indolent forms of systemic mastocytosis using flow cytometric immunophenotyping. A representative gating strategy and patient sample are presented in Figure 3.

Table 2 summarizes the percentage of mast cells, the expression of CD25 and CD2 markers, and the proportion of eosinophils identified in each case.

**Table 2:** Immunophenotypic characteristics assessed by flow cytometry in eight patients with systemic mastocytosis, including the percentage of mast cells, CD2 and CD25 expression, and eosinophil count

Patient	Mast Cells (%)	CD25 (%)	CD2 (%)	Eosinophils (%)
Case 1 – aggressive SM	0.16	21	33	2.0
Case 2 – aggressive SM	3.8	90	0.9	2.9
Case 3 – indolent SM	0.29	38	49	1.7
Case 4 –SM-AHN	0.04	71	58	2.0
Case 5 – indolent SM	1.0	7	9	17.0
Case 6 – indolent SM	0.1	95	64	1.9
Case 7 – aggressive SM	1.6	98	12	3.6
Case 8 – aggressive SM	8.0	98	37	2.3

SM, systemic mastocytosis; SM-AHN, systemic mastocytosis associated with hematological malignancy.

Despite the generally low percentage of clonal mast cells detected by flow cytometry (ranging from 0.04% to 8%), immunophenotypic evaluation based on CD25 and CD2 expression reliably confirmed aberrant mast cell populations in all eight cases (Table 2).

## DISCUSSION

This study provides a comprehensive overview of clinical, biochemical, and morphological variables in patients with SM in Romania, with a particular focus on sex-based differences, disease subtypes, and treatment response. The results shed light on several important aspects of SM pathophysiology and highlight some of the current challenges in patient management.

A clear female predominance was observed in the study cohort, aligning with prior reports that suggest systemic mastocytosis tends to affect women more frequently than men [17,18]. While the age at diagnosis did not differ significantly between sexes, the current age was statistically higher in females, which may reflect differences in disease progression or survival. Tryptase levels, although not significantly different between sexes, were consistently elevated, with female patients showing median values above the minor WHO diagnostic criterion (20 ng/mL), a finding that may carry diagnostic and prognostic relevance [19].

Bone marrow infiltration was moderate across both groups, with a median of 15% mast cells per aggregate. This finding supports the importance of bone marrow assessment in SM classification, as highlighted by the WHO and ECNM criteria [20]. Interestingly, our data indicate an inverse correlation between SSM and both tryptase levels and bone marrow infiltration, suggesting that SSM may occur in patients with lower disease burden, a phenomenon also discussed in other studies [21].

Bone involvement remains a significant concern, with nearly 40% of male and female patients showing signs of osteopenia or osteoporosis. Despite being a recognized feature of SM, bone disease is frequently underdiagnosed due to limited access to DXA scanning, long waiting times, and patient reluctance [22]. These barriers hinder proper risk stratification and contribute to the undertreatment of patients.

Correlation analysis highlighted important patterns across SM subtypes. CM was associated with higher tryptase levels and bone marrow infiltration, but paradoxically, patients with CM used fewer symptomatic treatments or antihistamines. This might reflect a milder systemic symptom burden or a clinical underestimation of the impact of cutaneous manifestations [23]. Indolent SM showed a clear link to symptomatic treatment use, suggesting that although classified as 'indolent', this form still imposes a significant symptomatic burden. The inverse correlation with bone marrow infiltration and *KIT* D816V mutation aligns with earlier evidence of heterogeneity in indolent SM [24].

Smoldering SM showed consistent moderate correlations with hepatomegaly and splenomegaly, reinforcing its intermediate position between intermediate SM and advanced forms. The inverse relationship with tryptase and bone marrow infiltration may imply early-stage systemic involvement with substantial organomegaly but limited mast cell burden. On the other hand, aggressive SM was associated with higher use of cladribine and imatinib, lower tryptase levels, and increased hepatosplenomegaly, consistent with its aggressive phenotype and previously published survival data [25-27]. Imatinib was used as a last resource in the treatment of aggressive mastocytosis before the approval of midostaurin.

Regarding the outcome analysis, patients treated with cytotoxic therapy had a median survival of only 204 days compared to over 450 days in those not receiving such treatment (Log-rank test, χ^2^ = 8.051; *P* = 0.005). While cytotoxic therapy is often used in advanced cases, the result may likely reflect indication bias – cytotoxic therapy being reserved for advanced disease, rather than the direct detrimental effect of treatment. The difference between groups may also reflect late treatment initiation, inadequate disease monitoring, or intrinsic resistance mechanisms. These results are in line with broader European survival data, which underscore the limited benefit of non-targeted therapies in aggressive SM [18,27-30].

Regarding the distribution of different SM subtypes, data from our cohort are compared with those from previous published reports in Table 3. In the Danish cohort, MIS was the most prevalent subtype, accounting for 47.6% of cases, followed by ISM at 27.1% [28]. The research from Sweden and the Mayo Clinic indicated ISM as the primary subtype, with rates ranging from as high as 72 % to 46 % respectively [28,31]. The cohort's findings also supported ISM as the predominant subtype for the Spanish population, at 65%, and reported WDS and SM anaphylaxis (each at 7%), which were not mentioned in other studies. The Mayo Clinic research highlighted that SM-AHN was more prevalent (40%), compared to lower rates in Denmark (11%), Sweden (18%), and Spain (12%) [18,28,31]. Regarding incidence rates, the Danish studies estimated SM occurrence between 0.74 and 2.77 cases per 1,000 individuals, while Sweden reported a rate of 1.56 per 1,000. Spain focused more on genetic markers rather than incidence figures alone [18,28,31]. In Swedish cases, the Charlson Comorbidity Index (CCI) indicated a burden of comorbidities, which underlines the importance of specialized care [28]. The research results highlight differences in the occurrence of mastocytosis across regions, which are likely influenced by diagnostic workflows and referral facilities, as well as a potential genetic predisposition specific to each population.

**Table 3:** Comparison of mastocytosis subtypes across the Danish, Mayo Clinic, Swedish, Spanish, and Romanian cohorts

Subtype	Danish Cohort (*n* = 1627)	Mayo Clinic Cohort (*n* = 342)	Swedish Cohort (*n* = 2040)	Spanish Cohort (*n* = 113)	Romanian Cohort (*n* = 162)
ISM	27.11%	46%	72%	65%	53.1%
SM-AHN	11%	40%	18%	12%	8%
ASM	0.25%	12%	8%	5%	3.7%
MCL	0.43%	1%	2%	5%	0%
MIS	47.57%	N/A	N/A	N/A	N/A
CM	13.64%	N/A	Included in SM	N/A	17.3%
WDSM	N/A	N/A	N/A	6%	N/A
SM-ana	N/A	N/A	N/A	6%	N/A
SSM	N/A	N/A	N/A	N/A	5.6%

SM, systemic mastocytosis; ISM, indolent systemic mastocytosis; SM-AHN, systemic mastocytosis with associated hematological neoplasm; MCL, mast cell leukemia; MIS, mastocytosis in the skin; CM, cutaneous mastocytosis; WDSM, well-differentiated systemic mastocytosis; SM-ana, systemic mastocytosis with recurrent anaphylaxis; SSM, smoldering systemic mastocytosis.

Findings from the Danish study and the Mayo Clinic report by Ken-Hong Lim *et al*. suggest that the incidence of SM has seen an increase in numbers, particularly because of the refined diagnostic tools [18,31]. Additional contributing factors include increased awareness of the disease and the emerging specialized referral centers, which now provide a more precise approach to patients likely to have such a diagnosis. Nevertheless, there is still room for improvement, as sometimes there is an incomplete data capture setting, mostly due to inconsistent coding of the diagnosis and difficult subtype classification in some cases.

Factoring in all of the above, there is a clear shift in the epidemiological landscape, with improved detection rates and a continuous drive for a more precise disease classification scheme, harmonized among national health systems. Moreover, emerging data describing the quality of life highlight the higher comorbidity burden and emphasize the need for specialized care and long-term monitoring strategies.

## CONCLUSION

This study highlights key demographic, molecular, and clinical features of systemic mastocytosis in a Romanian cohort, emphasizing sex-based differences, disease burden, and treatment response. The findings underscore the importance of accurate subtyping and molecular profiling in SM, as well as the need for timely access to diagnostic tools and effective, targeted therapies.

SSM and ASM subtypes demonstrated distinct correlations with organomegaly, bone marrow burden, and biochemical markers, reinforcing the necessity for individualized management strategies. The inverse correlation between tryptase and advanced disease manifestations in ASM and SSM raises important considerations for risk stratification and follow-up algorithms.

Most critically, survival analysis indicates that conventional cytotoxic therapies are insufficient in improving outcomes, possibly due to delayed initiation or lack of molecular targeting. These data support the international shift toward TKI-based therapies and early molecular assessment, aligning with findings from recent large-scale epidemiological and therapeutic studies.

In Romania, improved awareness, systematic implementation of WHO/ECNM guidelines, and better access to diagnostic and therapeutic advances are needed to optimize care for patients with SM.

Flow cytometric immunophenotyping confirmed the presence of aberrant mast cells in all patients with systemic mastocytosis, with CD25 being more frequently expressed than CD2, aligning with findings from recent literature. Notably, immunophenotyping proved valuable not only in advanced cases but also in indolent forms, particularly when peripheral blood tests were insufficient to meet WHO diagnostic criteria. However, the percentage of mast cells identified by flow cytometry should not be used as a surrogate for histological bone marrow infiltration, as it does not reflect mast cell morphology or tissue involvement.

The technique enables multiparametric analysis and quantification of surface marker expression, and preliminary observations suggest that higher levels of CD25 and CD2 expression may be correlated with disease aggressiveness. Due to the limited number of patients in this study, statistical confirmation of this association was not feasible, highlighting the need for multicenter studies in this rare disease. Looking forward, the use of immunophenotyping to evaluate treatment response—especially in patients receiving midostaurin—may support the detection of minimal residual disease, a concept not yet established in systemic mastocytosis but already integrated into the management of other hematologic malignancies.

## Data Availability

Further data is available from the corresponding author upon reasonable request.
